# Glycodeoxycholic acid as alternative treatment in 3β-hydroxy-Δ5-C_27_-steroid-oxidoreductase: a case report

**DOI:** 10.3389/fped.2024.1418963

**Published:** 2024-06-28

**Authors:** S. Majait, F. M. Vaz, E. Marleen Kemper, A. H. Bootsma, A. K. Groen, M. Nieuwdorp, Maarten R. Soeters

**Affiliations:** ^1^ Department of Pharmacy and Clinical Pharmacology, Amsterdam UMC Location University of Amsterdam, Amsterdam, Netherlands; ^2^ Department of Clinical Chemistry and Pediatrics, Laboratory Genetic Metabolic Diseases, Emma Children’s Hospital, Amsterdam UMC Location University of Amsterdam, Amsterdam, Netherlands; ^3^ Inborn Errors of Metabolism, Amsterdam Gastroenterology Endocrinology Metabolism, Amsterdam, Netherlands; ^4^ Core Facility Metabolomics, Amsterdam UMC Location University of Amsterdam, Amsterdam, Netherlands; ^5^ Department of Experimental Vascular Medicine, Amsterdam University Medical Center, Amsterdam, Netherlands; ^6^ Department of Vascular Medicine, Amsterdam UMC Location University of Amsterdam, Amsterdam, Netherlands; ^7^ Department of Endocrinology and Metabolism, Amsterdam UMC Location University of Amsterdam, Amsterdam, Netherlands

**Keywords:** 3β-HSD, bile acid defect, glycine-conjugated deoxycholic acid, FXR ligand, postprandial

## Abstract

**Background:**

3β-hydroxy-Δ5-C27-steroid-oxidoreductase (3β-HSD) deficiency is a bile acid synthesis disorder that leads to the absence of normal primary bile acids and the accumulation of abnormal bile acids. This results in cholestatic jaundice, fat-soluble vitamin deficiency, acholic or fatty stools and failure to thrive. Bile acid supplementation is used to treat 3β-HSD-deficiency and its symptoms.

**Methods:**

This report details the case of a 28-year-old woman diagnosed with 3β-HSD-deficiency, who was treated with glycine-conjugated deoxycholic acid (gDCA).

**Results:**

gDCA treatment successfully restored normal bile acid levels, improved body weight by reducing fat malabsorption, and was well-tolerated with no observed liver problems or side effects.

**Conclusions:**

As a potent FXR ligand, gDCA might exert its action through FXR activation leading to bile acid synthesis regulation.

## Introduction

3β-hydroxy-Δ5-C_27_-steroid-oxidoreductase (3β-HSD) deficiency is a bile acid synthesis disorder that is treated by bile acid supplementation (1). The enzyme 3β-HSD catalyzes the second step in the classic bile acid synthesis pathway. This deficiency is the most common inborn error of bile acid synthesis. 3β-HSD deficiency leads to the absence of normal primary bile acids and the accumulation of abnormal bile acids. Affected individuals present at birth or in early childhood with cholestatic jaundice, fat-soluble vitamin deficiency, acholic or fatty stools (steatorrhea) and failure to thrive.

Bile acid supplementation exerts negative feedback on bile acid synthesis via the Farnesoid X receptor (FXR) which prevents accumulation of toxic intermediates while restoring bile flow and the gastrointestinal uptake of fat and fat-soluble vitamins (2, 3). Deoxycholic acid (DCA) is the secondary bile acid of cholic acid (CA) which is formed by bacterial 7-dehydroxylation in the gut. It is a normal metabolite that is abundant in the human circulation with similar function as the primary bile acids (4). DCA is a good ligand for FXR like the primary bile acids CA and chenodeoxycholic acid (CDCA) (2). The majority of the human bile acid pool is in its conjugated form throughout the enterohepatic cycle, predominantly conjugated to glycine.

Therefore, we decided to treat our patient with glycine-conjugated DCA (gDCA). We had previously gained experience using gDCA in another project (5), which made it a well-suited choice for this case. The experimental nature of this treatment was discussed with the patient and her adoptive parents. The use of gDCA as therapy for 3β-HSD deficiency for this patient was reported to the Dutch Health Care Inspectorate before starting therapy (http://www.igz.nl/english, last accessed February 23, 2024).

## Case presentation

This case report details the case of a woman with 3β-hydroxy-Δ5-C27-steroid dehydrogenase (3β-HSD) deficiency, who was initially seen at the age of 4 at the Amsterdam Medical Center (AMC). She was born in India and adopted by Dutch parents at the age of 1 without knowledge of existing family. At the time of presentation, she was dystrophic with concomitant steatorrhea and deficiencies of fat-soluble vitamins. These symptoms were central in her medical history and therefore a malabsorption syndrome was suspected although a definitive diagnosis could not be made.

Initially her condition was attributed to tropical pancreatitis, due to possible earlier bouts of viral pancreatitis, whereas her short-stature was also partly explained by her Indian origin. During her treatment with hypercaloric nutrition, pancreatic enzymes and vitamin A, D, E and K, she was initially quite well. Her growth rate remained stable (−3.5< SD < −3.0). At the age of 11, she was finally diagnosed with 3β-HSD based on aberrant bile acid analysis. Genetic evaluation showed homozygosity for a nonsense mutation (Y12*) in exon 1 of the HSD3D7 gene. From that moment onwards she was treated with chenodeoxycholic acid (CDCA) (Chenofalk®) 5 mg/kg/day after which the steatorrhea ceased and her growth improved. In 2008, after three years of treatment, it was decided to replace CDCA with CA 250 mg twice daily because of presumed CDCA-related hepatotoxicity. In 2013, CA received European market authorization to be used in bile acid synthesis defects amongst which 3β-HSD-deficiency. Hereafter, the costs for CA treatment rose from ∼€14.000 to ∼€201.600 per year. Therefore, no affordable treatment was available and we decided to treat the patient with gDCA.

### Clinical assessment

To monitor the response to gDCA therapy, we analyzed glucose levels, liver function, vitamin status and hemostasis tests (Table 1). Also, we performed fecal fat balance tests. After cessation of CA therapy, our patient lost weight, experienced many complaints such as muscle weakness, muscle cramps and discolored feces. This weight loss was due to steatorrhea (0.14 gram fat per gram feces). The patient also described a notable tendency towards high-fat foods. To minimize potential side effects and ensure efficient bile acid pool retention, we initiated gDCA therapy gradually. Initially, she regained weight. However, based on clinical symptoms, we ultimately increased the dose to 4 times 10 mg/kg per week upon which she became symptom-free. We then lowered and kept the dose on 3 times 10 mg/kg per week. Also her weight continued to increase with negligible steatorrhea (0.003 g of fat per gram feces). Liver function tests showed higher bilirubin levels on two occasions during gDCA therapy, but all other bilirubin checks on gDCA therapy were normal. Both ALT and AST increased and decreased again. Fat-soluble vitamins were normal with vitamin A and E being slightly higher during gDCA 4 times per week compared to the other dosages. Periodic liver ultrasound examinations showed no abnormalities like steatosis. In conclusion, gDCA therapy restored body weight by limiting steatorrhea, was well tolerated and did not cause liver abnormalities.

**Table 1:** A summary of bile acid treatment markers.

	Reference values	CA 2/day	Off therapy	gDCA 2/week	gDCA 3/week	gDCA 4/week	gDCA 3/week 5 years
Glucose (mmol/L)	4.1–5.6	4.2	4.8	4.2	4.9	4.8	5.2
Albumen (gr/L)	35–50	40	46	40	41	41	46
Creatinine (μmol/L)	65–95	58	49	58	47	58	55
BUN^a^ (mmol/L)	2.1–7.1	2.7	2.6	2.7	1.6	3	3.2
Bilirubin-total (μmol/L)	0–17	4	8	13	14	11	20
Bilirubin-direct (μmol/L)	0–7	2	5	7	6	4	8
AST^a^ (U/L)	0–40	25	26	34	31	23	25
ALT^a^ (U/L)	0–34	12	23	42	26	23	32
Total cholesterol (mmol/L)	<5.0	3.89	3.45	3.89	2.75	3.88	3.11
HDL^a^ Cholesterol (mmol/L)	>1.0	1.23	0.96	1.23	0.73	1.22	1.04
LDL^a^ cholesterol (mmol/L)	<3.0	2.36	1.57	2.36	1.4	2.13	1.35
Triglycerides (mmol/L)	0.5–2	0.66	2.04	0.66	1.37	1.18	1.59
Vitamin A (μmol/L)	1.2–2.7	1	1.9	1.7	2.4	1.8	1.8
Vitamin D (nmol/L)	>50	102	44	85	80	73	28
Vitamin E (μmol/L)	15–35	23.9	25.4	24.6	33.1	34.8	23.2
PT^a^ (secs)	9.7–11.9	12.6	13.5	N.A^1^	12.7	11.8	11.8
Fecal fat (gram/gram feces)			0.003		0.14	0.003	
Weight (kg)		36	33	37	38	40	40

^a^ BUN, blood urea nitrogen; AST, aspartate aminotransferase; ALT, alanine aminotransferase; HDL, high density lipoprotein; LDL, low density lipoprotein; PT, prothrombin time; N.A., not analyzed.

### Treatment

**Figure 1:**
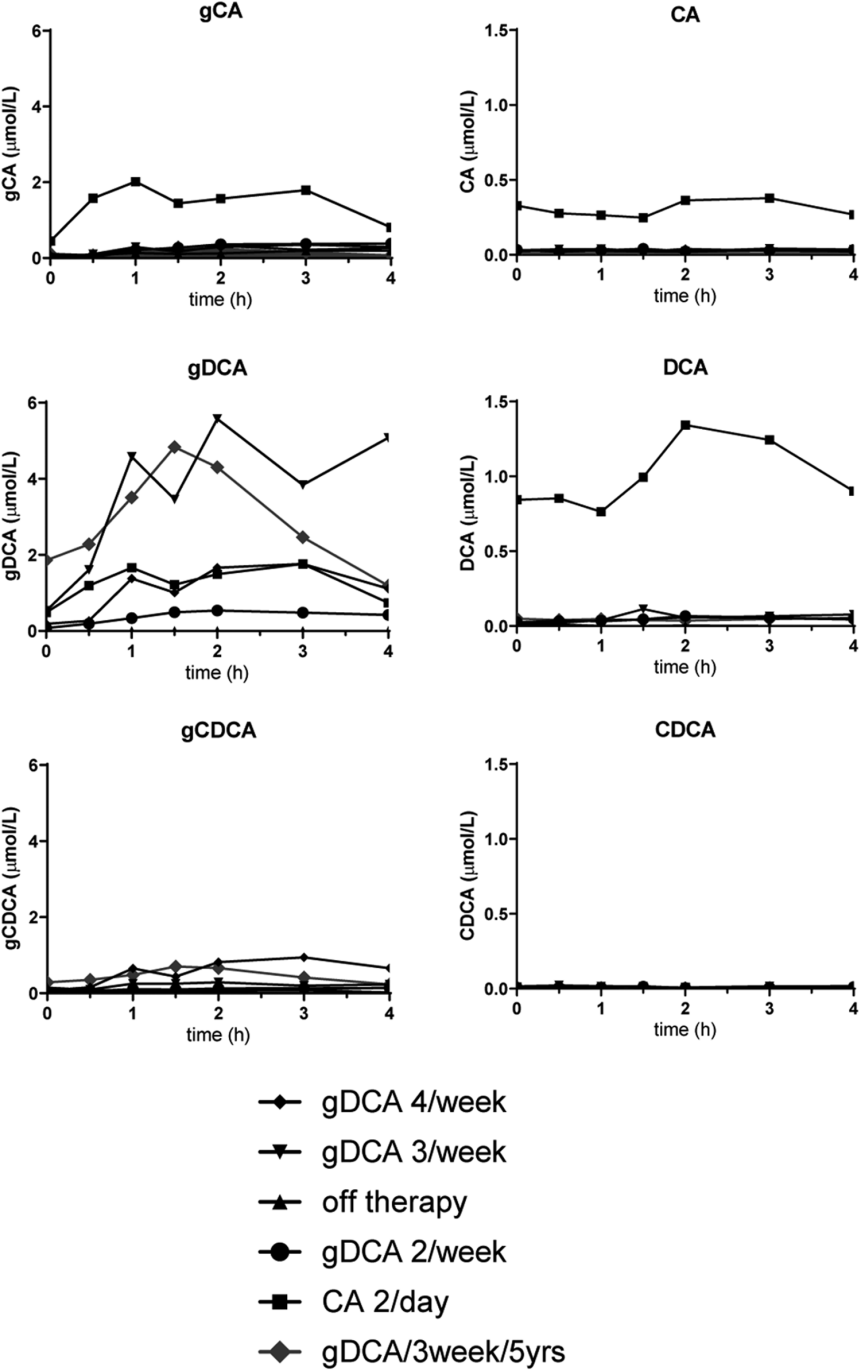
Meal test curves of conjugated and unconjugated bile acids. gCA, glycocholic acid; CA, cholic acid; gDCA, glycodeoxycholic acid; DCA, glycodeoxycholic acid; gCDCA, glycochenodeoxycholic acid; CDCA, chenodeoxycholic acid.

**Figure 2:**
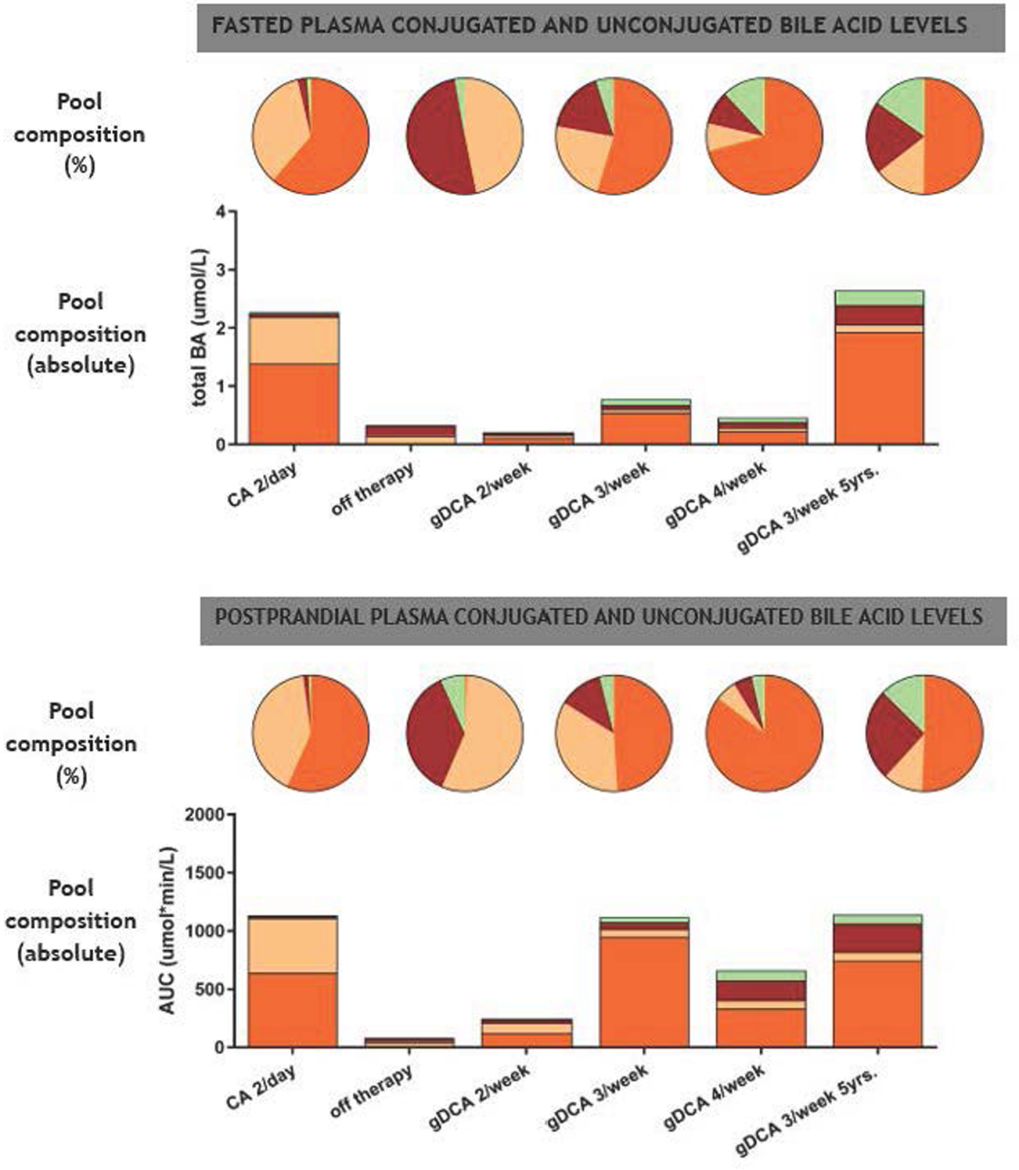
Fasting and postprandial composition of plasma conjugated and unconjugated bile acid levels. Pie charts indicate bile acid pool composition in percentage whereas bar charts indicate absolute bile acid pool composition. BA, bile acid; CA, cholic acid (beige); DCA, deoxycholic acid (orange); UDCA, ursodeoxycholic acid (green); CDCA, chenodeoxycholic acid (red).

We studied our patient after gDCA 10 mg/kg twice per week, three times per week and four times per week. To allow for comparison, we also studied her on the usual dose of CA (250 mg twice daily) and off therapy. We allowed ∼2 months equilibration of a new therapy before test days. To investigate bile acid (BA) flow, we analyzed postprandial plasma levels after standardized liquid mixed meals (Figures 1, 2). The method of the mixed meal test is described in the Supplementary Material (6).

Figure 1 shows the postprandial curves of the plasma glycine-conjugated and unconjugated bile acids. Conjugated bile acids were more abundant compared to unconjugated bile acids. gDCA was relatively high during all therapies including CA administration. In contrast, unconjugated DCA and CA was only seen during CA treatment. Both conjugated and unconjugated CDCA were low with the former being more abundant during gDCA 4 times a week therapy. Figure 2 shows fasted and postprandial plasma bile acid levels expressed as percentage of the pool and as absolute values. After the start of gDCA therapy, plasma bile acid levels were still low, and DCA contribute the most to the bile acid pool. Increasing the dose further eventually led to a more or less evenly distributed bile acid composition (Figure 2, upper panel).

Analogous to the fasting plasma samples, postprandial bile acids under CA therapy consisted of DCA and CA predominantly, with low plasma bile acid levels (CA and CDCA) after withdrawal of therapy. Starting and increasing the gDCA dose further led to a relative well-distributed postprandial bile acid composition (Figure 2, lower panel).

### Follow-up

We re-evaluated the patient's bile acid function with a mixed meal tolerance test after five years of treatment. Laboratory findings and weight remained stable (Table 1). Fasted and postprandial bile acid levels remained comparable during five years of gDCA 3/week treatment (Figures 1, 2).

## Discussion

A diverse spectrum of point mutations, small insertions and deletions in the HSD3B7 gene of affected individuals are present in homozygous and compound heterozygous forms of 3β-HSD-deficiency (7). Bile acid supplementation exerts negative feedback on bile acid synthesis preventing accumulation of toxic intermediates while restoring bile flow and the gastrointestinal uptake of fat and fat-soluble vitamins. Here, CA treatment has historically been used because it has been deemed safe and effective (4). In our patient, cessation of bile acid therapy induced a relapse towards the characteristic symptoms of the disease including weight loss, fatty stools and accumulation of bile acid intermediates. Liver function tests or vitamin status did not show striking results, which may have been due to the fact that withdrawal of therapy was only ∼2 months. We allowed the patient to be off-therapy for such a long period, since previously she went undiagnosed for ∼11 years. After re-initiation of therapy, a gradual improvement occurred in terms of subjective complaints, fatty stools, weight and aberrant peaks of the bile acid chromatogram. gDCA treatment was maintained and the patient has remained in good health for the past five years.

Most primary and secondary bile acids exert negative feedback on endogenous bile acid synthesis via FXR (2, 3). Different bile acids have different affinity for FXR, where CDCA is the most potent followed by DCA and CA (2). UDCA is a poor FXR ligand (8). DCA makes up a large part of the human bile acid pool in healthy individuals (3, 9). Furthermore, DCA has been shown to suppress bile acid biosynthesis in humans to the same degree as CDCA as judged from the suppression of plasma levels of 7*α*-hydroxy-4-cholesten-3-one, a surrogate marker of CYP7A1 activity (9). In our patient, CA therapy resulted in a bile acid pool of which the majority consisted of DCA, which can be explained by bacterial conversion of CA in DCA. Notably, FXR is an intra-nuclear receptor and it has been proposed that unconjugated bile acids are more likely to inhibit FXR activity compared to conjugated bile acids. It has also been shown that glycine conjugated bile acids such as gDCA are capable to reduce bile acid biosynthesis (8).

The specific factors influencing the patient's bile acid pool composition remain unclear. Bacterial dehydroxylation of CA results in DCA during CA therapy and in normal circumstances (3). However, during gDCA therapy, CA, CDCA and UDCA all emerged. Besides ileal uptake of gDCA, a small part of gDCA will reach the distal intestine and is deconjugated. Then, CA is probably derived from DCA via from hepatic 7*α*-hydroxylation as shown in rats (10). CDCA may be produced by *Bacteroides* strains that reduce CA to CDCA (11). The fact that CA therapy did hardly produce CDCA may be due to the fact that probably very low levels of CA reached the distal gut.

Also, the bile acid profile of our patient improved remarkably during gDCA therapy compared to CA therapy. This might be explained by the fact that glycine-conjugated bile acids are absorbed more easily compared to unconjugated bile acids. However, we did not quantify atypical 3β-hydroxy-Δ5-bile acids that are the signature metabolites for this bile acid synthesis disorder.

Safety issues and side effects (e.g., elevated liver transaminases and gastrointestinal complaints) occur frequently in prolonged bile acid supplementation as frequently seen in inherited bile acid synthesis disorders (12). In these conditions, the mainstay of treatment is bile acid therapy to supplement missing bile acids, but also to inhibit bile acid synthesis and thereby preventing the accumulation of toxic intermediates (12). When side effects occur, dose reduction of oral bile acids is warranted. A possible manner to prevent these adverse effects is to administer bile acids in a conjugated form since conjugation to glycine or taurine reduces bile acid cytotoxicity (13, 14). Moreover, under physiological circumstances, bile acids are excreted by the gallbladder in their conjugated form which supports the idea of conjugated bile acid supplementation.

No signs of side-effects in our patient, nor in the healthy volunteers that we have treated with gDCA are found so far (5). The supposed hepatotoxic side-effects of bile acid treatment have not been very well characterized (15, 16). Jaundice, often self-limiting and giant cell hepatitis have been mentioned in both children and adults (15, 17). Moreover, the side effects of CDCA therapy have primarily been shown in the bile acid biosynthesis defect cerebrotendinous xanthomatosis (CTX) in which cholestasis may occur as part of the disease (17). DCA has been studied in relation to cholesterol absorption and metabolism (18). Other formulations of DCA have potential clinical use in fat reduction (subcutaneous injection), anti-cancer activity and drug delivery (19).

In this case report, we detail our clinical application of glycine conjugated deoxycholic acid as treatment for 3β-HSD-deficiency in terms of physiology, efficacy and safety. We acknowledge that this “*n* = 1” treatment needs further study despite the fact that the number of patients with inherited bile acid disorders is very limited. Therefore, we also advise to monitor liver function during bile acid therapy. We used ultrasound to examine focal liver abnormalities and steatosis, but did not monitor the presence of fibrosis. Larger trials with conjugated bile acids in the field of inherited bile acid disorders may be warranted but are almost impossible due to limited numbers of patients. Indeed, most evidence is anecdotal and based on expert experience.

Another problem is the European market authorization of CA (and later CDCA) due to orphan drug legislation (EU/3/02/127 | European Medicines Agency (ema.europa.eu) and EU/3/14/1406 | European Medicines Agency (ema.europa.eu), last accessed March 13, 2024) and the enormous costs. Actually, this is not different from the United States, where CA was approved on March 17th 2015 by the FDA for treatment of bile acid synthesis disorders due to single enzyme defect with the same pricing issue [Drug Trials Snapshot: CHOLBAM (bile acid synthesis disorders) | FDA, last accessed March 13, 2024].

It is inevitable that conjugated bile acid therapy will experience the same price increase as CA and CDCA. However, from a clinical, patient and societal point of view, we deemed it important to share our clinical experience with glycine conjugated DCA in our patient with 3β-HSD-deficiency.

## Data Availability

The raw data supporting the conclusions of this article will be made available by the authors, without undue reservation.
