# Supplementary material for: Glycodeoxycholic acid as alternative treatment in 3β-hydroxy-Δ5-C27-steroid-oxidoreductase: a case report
Source: Front Pediatr. 2024 Jun 28;12:1418963. doi: 10.3389/fped.2024.1418963 (PMC11239425; doi:10.3389/fped.2024.1418963)
Supplement: Supplementary file 1 [file Datasheet1.pdf]

Supplementary Material

Mixed meal test protocol

On study days, our patient was admitted at 07:30h to the Experimental and Clinical Research Unit (ECRU) of the AMC after an overnight fast. A cannula was inserted into an antecubital vein for blood sampling. This hand was kept in a heated hand box throughout the test to arterialize venous blood. At 09:30h, 3 blood samples were taken at 10-minute intervals for the determination of basal plasma glucose and insulin concentrations. At 10:00h, our patient consumed a standardized liquid mixed meal (Nutridrink, Nutricia, Zoetermeer, The Netherlands) containing ~25% of the daily energy requirements (6.25 kcal/kg). Hereafter, blood samples were obtained at 0, 30, 60, 90, 120, 180 and 240 minutes after the meal. Blood was collected into chilled tubes containing either EDTA or heparin as anticoagulant on ice and immediately centrifuged, and plasma was subsequently stored at –20° C until analysis. BAs were determined using a UPLC-tandem MS method to detect CA, CDCA, DCA and UDCA in their conjugated and unconjugated forms (19). We measured postprandial bile acid levels as a measure of postprandial bile flow and to further examine the bile acid pool composition.
